# Body weight, body composition and survival after 1 year: follow-up of a nutritional intervention trial in allo-HSCT recipients

**DOI:** 10.1038/s41409-019-0638-6

**Published:** 2019-08-27

**Authors:** K. J. Skaarud, M. B. Veierød, S. Lergenmuller, A. Bye, P. O. Iversen, G. E. Tjønnfjord

**Affiliations:** 1 Department of Haematology, Oslo University Hospital, Oslo, Norway; 2 Department of Nutrition, Institute of Basic Medical Sciences, University of Oslo, Oslo, Norway; 3 Oslo Centre for Biostatistics and Epidemiology, Department of Biostatistics, Institute of Basic Medical Sciences, University of Oslo, Oslo, Norway; 4 European Palliative Care Research Centre, Department of Oncology, Oslo University Hospital and Institute of Clinical Medicine, University of Oslo, Oslo, Norway; 5 Department of Nursing and Health Promotion, Faculty of Health Sciences, Oslo Metropolitan University, Oslo, Norway; 6 Institute of Clinical Medicine, University of Oslo, Oslo, Norway; 7 K.G. Jebsen Centre for B-Cell Malignancies, University of Oslo, Oslo, Norway

**Keywords:** Haematological cancer, Randomized controlled trials

## Abstract

The role of body weight change in survival among recipients of hematopoietic stem-cell transplantation is controversial. We assessed the effect of optimizing energy and protein intake on 1-year survival, body weight and body composition, and the effect of body weight and body composition on 1-year survival in 117 patients (57 intervention, 60 control) in a randomized controlled trial. Cox regression was used to study effects of the intervention, weight and body composition on death, relapse, and nonrelapse mortality (NRM). We found no significant effect of intervention versus control on death hazard ratio (HR) 1.05, 95% confidence interval (CI) 0.54−2.04, *p* = 0.88), relapse (HR 1.15, 95% CI 0.48−2.27, *p* = 0.75), and NRM (HR 0.95, 95% CI 0.39−2.28, *p* = 0.90). Body weight, fat-free mass index, body fat mass index and total body water changed over time (*p* < 0.001), similarly in both groups (0.17 ≤ *p* ≤ 0.98). In multivariable analyses adjusted for group, gender and age, HRs and 95% CIs per one kilo increase in weight were 1.03 (1.01−1.06) and 1.04 (1.01−1.08) for death and NRM after 1 year (*p* ≤ 0.02), respectively, and 1.08 (1.01−1.15) for relapse after 3 months (*p* = 0.02). In conclusion, weight gain is possibly due to fluid retention and is an indicator of a complication in HSCT, rather than a marker of improved nutritional status.

## Introduction

Upon admission for allogeneic hematopoietic stem-cell transplantation (allo-HSCT) many patients have lost body weight and some may even be malnourished [1, 2]. Weight loss often increases in the peritransplant period [3–6]. A study found increased risk of nonrelapse mortality (NRM) and inferior overall survival in patients with >10% weight loss compared with weight loss <10% [3], while an association between weight loss and survival was not found in another study [5]. One complicating factor when measuring body weight in the early phase of transplantation is the influence of capillary leak and fluid retention [7–9], which may mask weight loss. Therefore, measures of the different body composition compartments i.e. muscle and fat mass and total body water would be more relevant. To the best of our knowledge, no former study has measured body composition longitudinally after allo-HSCT and assessed the impact on survival.

Several studies have assessed the effect of nutritional interventions on body weight and survival in allo-HSCT recipients. A randomized controlled trial (RCT) of old date, compared total parenteral nutrition with an electrolyte-enriched solution [10], and a later RCT compared individually adjusted parenteral nutrition with an electrolyte-enriched solution [11]. Both studies reported improved body weight [10, 11], and the former found improved overall survival [10]. However, there are no data showing that parenteral nutrition is superior to enteral nutrition in allo-HSCT [12–14]. Furthermore, it is not certain whether nutritional support during hospitalization for allo-HSCT improves survival.

We previously reported results of an RCT investigating optimized energy and protein intake compared to routine hospital procedure for nutritional support on global quality of life and clinical outcomes 3 months post-HSCT. The intervention had no effect on the primary endpoint, global quality of life, or the main secondary outcomes, oral mucositis and acute graft-versus-host disease (aGVHD) [15]. The aim of this 1-year follow-up was to explore: (i) the effect of the intervention on death, relapse and NRM, (ii) changes in weight and body composition, and (iii) the effect of changes in weight and body composition on death, relapse and NRM.

## Patients and methods

### Patients

In total, 117 eligible patients (intervention: *n* = 57, control: *n* = 60) with a hematological malignancy ≥18 years of age, and able to provide informed consent received myeloablative conditioning before allo-HSCT were included in the study [15]. Data were collected from August 2010 to February 2017 at Oslo University Hospital, Norway. The study was approved by The Regional Committee for Medical and Health Research Ethics South East Norway (#S-09136c 2009/2115) and the Data Protection Supervisor, Oslo University Hospital, and registered at ClinicalTrials.gov, ID NCT01181076.

### Procedures

The procedures have been described in detail [15]. All patients received either (i) intravenous busulphan (four oral doses/day with target serum concentration of 900 ng/ml from days −7 to −4 prior to the transplantation) and intravenous cyclophosphamide (60 mg/kg body weight on days −3 and −2); or (ii) total body irradiation (1.3 Gy × 2 on days −8 to −4) combined with intravenous cyclophosphamide (60 mg/kg on days −3 and −2). Allografts were mobilized peripheral-blood stem cells or bone marrow cells from HLA-identical related or unrelated donors. GVHD prophylaxis was cyclosporine and methotrexate.

The nutritional intervention started when conditioning was initiated and continued until hospital discharge, and aimed at a daily energy intake of ≥126 kJ/kg body weight and a protein intake of 1.5−2.0 g/kg body weight [15]. Patients received routine hospital food and were encouraged to take energy-enriched and lactose-reduced snacks and oral supplements daily. A nasoenteric tube was inserted within 5 days after transplantation. Those unable to meet the energy target by the oral or enteral route received supplementary parenteral nutrition (PN). Oral, enteral and PN energy intake was monitored on a daily basis. After discharge, nutrition advice, oral nutritional supplements and enrichment powder were provided at the regular outpatient visits. The control group received routine hospital procedure for nutritional support. During hospitalization, the intervention group received significantly more (median (range)) energy (kJ/kg) and protein (g/kg) compared to a reference group for the control group, 131.9 (58.2−178.7), 1.1 (0.5−1.5) and 99.2 (50.2−139.8), 0.6 (0.4−1.0) (*p* < 0.001, *p* < 0.001), respectively [15].

### Definition and assessment of study outcomes

Power calculation was based on the trials initial primary endpoint as previously described [15]. Outcome variables in this follow-up study were death, relapse, and NRM 1 year post-HSCT, and body weight, fat-free mass index (FFMI) (including body cell mass, extracellular solids, extracellular water and intracellular water) and body fat mass index (BFMI) during the first year post-HSCT. One year post-HSCT was defined as the day the patients arrived for the regular 1-year visit. Death was defined as death from any cause, relapse was defined as bone marrow blasts >5% and NRM as death from any cause except from relapse. Weight was measured by a Tanita scale (BC-418 MA, Tanita Corp., Tokyo, Japan) to the nearest 0.1 kg. A correction factor of 0.1 kg was used to adjust for the weight of light clothing. Body composition was estimated using a single frequency bioimpedance analyzer (Tanita scale), operating at 50 kHz, with eight-point contact electrodes. FFMI and BFMI were calculated as fat-free mass and body fat mass (kg/m^2^). Body weight, FFMI and BFMI were assessed at inclusion before conditioning, at 3 and 6 weeks and 3, 6, 9 and 12 months post-HSCT. Weight 6 months before inclusion was self-reported using the Patient-Generated Subjective Global Assessment form [16]. FFMI was considered as low if <17 kg/m^2^ for men and <15 kg/m^2^ for women [17]. Based on a previous study BFMI was considered low if <2 kg/m^2^ for men and <4 kg/m^2^ for women [18]. The use of glucocorticoids was determined as the number of days for each patient on such treatment.

### Statistical analyses

Cox regression was used to study the effects of the treatment group (intervention versus control), weight, FFMI and BFMI on death, relapse and NRM, using the standard competing risk framework for relapse and NRM [19], while the Kaplan−Meier method was used to compare the probability of overall survival between the intervention and control group. We present hazard ratios (HRs) and 95% confidence intervals (CIs). Two patients had their allograft rejected and were censored at the time of the event when analyzing death, relapse and NRM. Those who rejected the allograft or relapsed had no further observations of weight and body composition. Body weight, FFMI, and BFMI were modeled as time-dependent continuous variables. In the multivariable analysis we adjusted for treatment group, gender, and age (categorized as <44 and ≥44 years). We conducted a sensitivity analysis for death, also adjusting for discharge status (not discharged/discharged from hospital stay) as a time-varying covariate. Additional adjustment for disease status (standard risk and high risk) (see Table 1), Hematopoietic Cell Transplantation-specific comorbidity index (low risk and intermediate/high risk) [20] and European Group for Blood and Marrow Transplantation score (low and intermediate/high) [21] did not change the results. The proportional hazard assumption was not fulfilled in the analysis of relapse, but when introducing a cut-off at 100 days the assumption was met. We also studied baseline body weight, FFMI and BFMI (all continuous) and weight change before baseline as a categorical variable (no weight loss, >0−<5% loss, 5−<10% loss, and ≥10% loss; the first category includes the patients that gained weight). Body weight, FFMI, BFMI, and total body water were analyzed with a linear mixed model for repeated measures using all-time points (baseline, 3 and 6 weeks and 3, 6, 9 and 12 months). We tested for interaction between group and time. Chi-square or Fisher exact test was used to study the use of glucocorticoid (yes, no) during the early phase (baseline to 6 weeks) in relation to weight change (no weight gain/weight loss versus weight gain). A *p* value < 0.05 was considered statistically significant. Analyses were performed with SPSS 26 (IBM Corp., Armonk, NY) and with R package bda, version 3.5.2 (R Core Team, Vienna, Austria).

**Table 1:** Clinical characteristics at inclusion (previously published [15])

Characteristics	Intervention	Control
	(*n* = 57)	(*n* = 60)
Age yr—median (range)	45 (19−65)	41 (18−62)
Female	20 (35)	25 (42)
AML	36 (63)	31 (51)
High risk first remission	23	22
After relapse, beginning of first relapse and in second remission	10	9
First remission standard risk	3	—
ALL	6 (10)	10 (17)
First remission high risk	3	7
Early first relapse, second remission	3	3
CML	2 (4)	7 (12)
Chronic phase	—	1
Accelerated phase	2	6
CMML	3 (5)	3 (5)
MDS	6 (11)	5 (8)
Other^a^	4 (7)	4 (7)
Donor		
HLA-identical sibling	17 (30)	13 (22)
HLA-identical unrelated	40 (70)	47 (78)
Stem-cell source		
Bone marrow	25 (44)	27 (45)
Peripheral-blood hematopoietic cells	32 (56)	33 (55)
Sex mismatch^b^	17 (30)	10 (17)
Positive CMV serology		
Donor	27 (47)	24 (40)
Recipients	45 (79)	43 (72)
Conditioning		
Busulphan + Cyclophosphamide	56 (98)	56 (93)
TBI + Cyclophosphamide	1 (2)	4 (7)
HCTI—CI risk groups^a^		
Low risk	42 (74)	45 (75)
Intermediate risk	8 (14)	10 (17)
High risk	7 (12)	5 (8)
EBMT score^a^		
0−3	33 (58)	36 (60)
4	14 (24)	14 (23)
5−7	10 (18)	10 (17)
Performance status ECOG		
0	55 (96)	54 (90)
1	2 (4)	6 (10)
BMI		
Underweight	2 (4)	4 (7)
Normal weight	31 (54)	27 (45)
Overweight	17 (30)	26 (43)
Moderately obese	4 (7)	3 (5)
Severely obese	3 (5)	0 (0)

Values are numbers (%) unless otherwise stated

*AML* acute myeloid leukemia, *ALL* acute lymphocytic leukemia, *CML* chronic myeloid leukemia, *CMML* chronic myelomonocytic leukemia, *MDS* myelodysplastic syndrome, *CMV* cytomegalovirus, *TBI* total body irradiation, *HCTI-CI* hematopoietic cell transplantation-specific comorbidity index, *EBMT* score European Group for Blood and Marrow Transplantation score, *ECOG* Eastern Cooperative Oncology Group

^a^An expanded list of baseline values for other diagnosis, EBMT score and HCTI-CI score is provided in Supplementary Table S1

^b^Sex mismatch was defined as female donor to male recipients

## Results

**Fig. 1:**
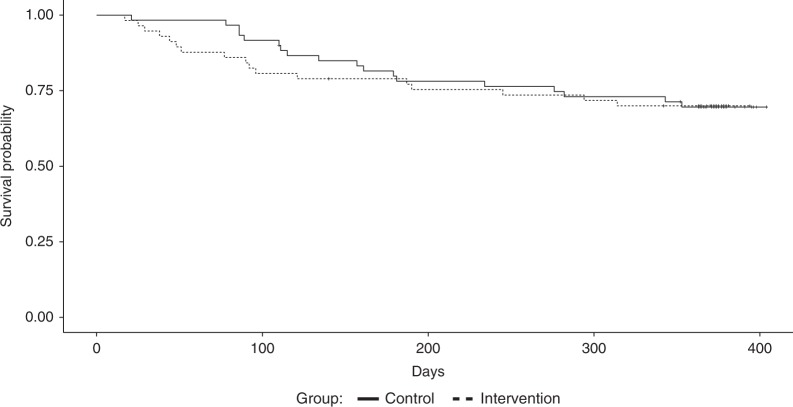
Kaplan−Meier plot for 1-year overall survival

Characteristics at inclusion of the 117 patients (intervention: *n* = 57, control: *n* = 60) are shown in Table 1 and Supplementary Table S1. In total, 35 (29.9%) patients died during 1-year follow-up (17 in the intervention and 18 in the control group). One-year overall survival was similar in the intervention and control group (*p* = 0.88) (Fig. 1). Twenty (17.1%) patients suffered a relapse (10 in both groups), and NRM included 20 (17.1%) patients (9 and 11, respectively). Five patients who relapsed were still alive at 1 year. Results of analysis of death, relapse, and NRM within 1-year post-HSCT are shown in Table 2. We found no significant effect of the nutritional intervention on any of the three outcomes (0.75 ≤ *p* ≤ 0.90, Table 2).

**Table 2:** Hazard ratios (HRs) and 95% confidence intervals (CIs) for treatment group, body weight, fat-free mass index and body fat mass index, and risk of death, relapse and nonrelapse mortality

			Univariable		Multivariable^a^	
Variables	*N*	Cases	HR (95% CI)	*p* value	HR (95% CI)	*p* value
Death						
Group^b^	117	35	1.05 (0.54−2.04)	0.88		
Weight (kg)	117	35	1.03 (1.00−1.05)	0.02	1.03 (1.01−1.06)	0.01
FFMI (kg/m^2^)	115	35	1.05 (0.94−1.17)	0.41	1.07 (0.91−1.26)	0.42
BFMI (kg/m^2^)	115	35	1.13 (1.01−1.26)	0.03	1.19 (1.05−1.35)	0.01
Relapse						
Group^b^	117	20	1.15 (0.48−2.27)	0.75		
Weight (kg) ≤ 100 days^c^	117	20	0.98 (0.94−1.03)	0.48	0.96 (0.91−1.02)	0.17
Weight (kg) > 100 days^c^			1.07 (1.02−1.13)	0.01	1.08 (1.01−1.15)	0.02
FFMI (kg/m^2^) ≤ 100 days^c^	115	20	0.95 (0.77−1.16)	0.59	0.82 (0.61−1.09)	0.17
FFMI (kg/m2) > 100 days^c^			1.34 (1.03−1.75)	0.03	1.44 (1.00−2.06)	0.05
BFMI (kg/m^2^)	115	20	1.02 (0.86−1.20)	0.83	1.06 (0.89−1.27)	0.50
NRM						
Group^b^	117	20	0.95 (0.39−2.28)	0.90		
Weight (kg)	117	20	1.03 (1.00−1.06)	0.06	1.04 (1.01−1.08)	0.02
FFMI (kg/m^2^)	115	20	1.07 (0.92−1.23)	0.40	1.13 (0.90−1.41)	0.30
BFMI (kg/m^2^)	115	20	1.12 (0.97−1.29)	0.11	1.18 (1.01–1.39)	0.04

*FFMI* fat-free mass index, *BFMI* body fat mass index, *NRM* nonrelapse mortality

^a^Adjusted for treatment group, gender and age

^b^Intervention versus control

^c^The effect changed over time (nonproportional hazards) and follow-up was split into two periods ≤100 and >100 days

**Fig. 2:**
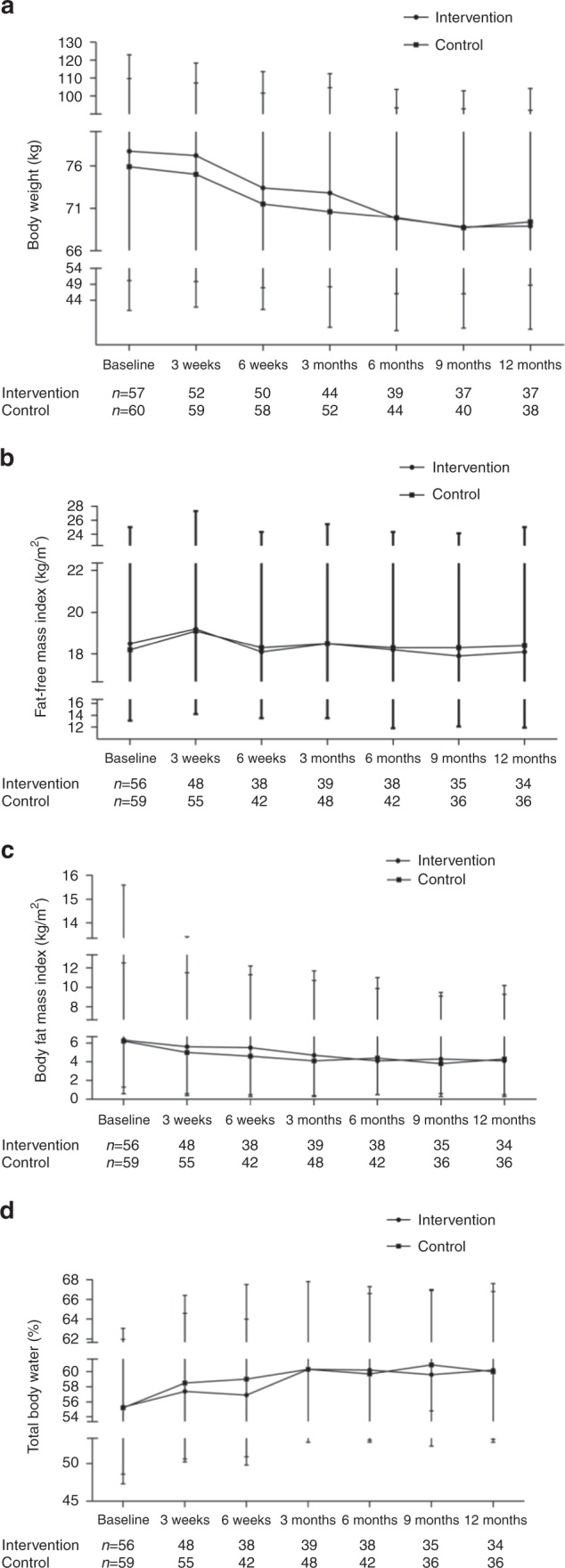
Mean values and standard errors for: **a** body weight (kg), **b** fat-free mass index (kg/m^2^), **c** body fat mass index (kg/m^2^) and **d** total body water (%) during 1-year follow-up

Significant changes were observed in body weight, FFMI, BFMI and total body water in both the intervention and control group during the 1-year follow-up (*p* < 0.001 for all), but with no significant differences between the groups (0.17 ≤ *p* ≤ 0.98) as shown in Fig. 2a–d and Supplementary Table S2. Also, no significant interaction was found between group and time, i.e. the effect of time was similar in both groups (0.12 ≤ *p* ≤ 0.65). Mean body weight and BFMI decreased during the first 6 months, and then remained fairly stable throughout the 1-year follow-up (Fig. 2a, c). Mean FFMI and total body water increased initially, while only FFMI decreased before stabilizing (Fig. 2b, d).

Table 2 shows that the effects of weight, FFMI and BFMI on 1-year death, relapse and NRM were quite similar in univariable and multivariable analysis. Here we report multivariable results. For each kg increase in body weight the risk of death increased with 3% (*p* = 0.01) and the risk of NRM with 4% (*p* = 0.02). No significant association was found between weight and the risk of relapse over the whole period (*p* = 0.57); however, due to lack of proportional hazards, follow-up was split. No significant association was found between weight and risk of relapse during the first 100 days (*p* = 0.17), while a significant effect was found after 100 days (6% increased risk per kg increase in weight, *p* = 0.02). FFMI was not significantly associated with death (*p* = 0.42) or NRM (*p* *=* 0.30). FFMI was not significantly associated with relapse before 100 days (*p* = 0.17), and borderline significant after 100 days (*p* = 0.05). BFMI was significantly associated with death (*p* = 0.01) and NRM (*p* = 0.04), while no association was found with relapse (*p* = 0.50) (Table 2). Adjustment for discharge status did not change the associations between weight, FFMI, BFMI and death (0.01 ≤ *p* ≤ 0.50) (Supplementary Table S3).

We also assessed the effects of weight, FFMI and BFMI at baseline on death, relapse and NRM 1-year post-HSCT with similar results in univariable and multivariable analyses except for BFMI who changed from nonsignificant in the univariable to significant in the multivariable analysis (Supplementary Table S4a), the multivariable *p* values are reported here. We found no effects of baseline weight and FFMI (0.14 ≤ *p* ≤ 0.97), while baseline BFMI was significantly associated with death (*p* = 0.04) and increased NRM (*p* = 0.03), but not with relapse (*p* = 0.83). Moreover, no significant association was found between weight change the last 6 months before allo-HSCT and death, relapse and NRM 1-year post-HSCT (0.37 ≤ *p* ≤ 0.93) (Supplementary Table S4b).

Twenty-one (36.8%) patients in the intervention and 24 (40.0%) in the control group received glucocorticoid treatment in the early post-transplant period. Median (range) numbers of days with glucocorticoids were 25 (2−78) and 34 (8−88), respectively. We found a significant association between glucocorticoid usage and weight change from baseline to 6 weeks post-transplant; 25.7% among those treated with glucocorticoids and 5.5% among those not treated with glucocorticoids gained weight (*p* = 0.004), but not from baseline to 3 weeks (corresponding percentages were 33.7% and 31.3%, respectively, *p* = 0.85, Supplementary Table S5).

## Discussion

The present study yielded three main results. Firstly, we found no significant difference between the intervention group with optimized energy and protein intake and the control group in risk of death, relapse and NRM within 1 year of allo-HSCT. Secondly, body weight and body composition changed significantly during 1-year follow-up, and the changes were similar in both groups. Thirdly, weight gain increased the risk of death and NRM during 1-year follow-up and the risk of relapse after 100 days (i.e. 3 months). We found the same association between weight gain and death when adjusting for discharge status.

There might be several explanations for why optimized energy and protein intake was not superior to routine hospital procedure for nutritional support on 1-year survival. Firstly, the targeted 126 kJ/kg and 1.5−2.0 g protein/kg calculated at baseline may be too low in the intense catabolic phase following allo-HSCT [22]. However, a higher energy intake may be difficult to achieve without a risk of fluid overload in the early post-transplantation period. Secondly, we cannot ignore the possibility of no differences in energy and protein intakes between the intervention group and control group, since oral energy intake was not monitored in the control group to avoid unintended focus on nutrition. However, we monitored energy and protein intakes in a reference group prior to the study and found no significant differences between the controls and the reference group in energy and protein intakes derived from glucose, enteral and parenteral nutrition. Moreover, total energy and protein intakes were significantly lower in the reference group than in the intervention group [15]. Thirdly, the effect of enteral nutrition (EN) versus PN on survival has been debated. All the intervention patients received a PN supplement in addition to EN to achieve the targeted energy requirement [15]. Similar to the present study, two observational studies found no effect of EN combined with PN on body weight compared to PN alone [23, 24], but 3 months overall survival was better in the EN group compared to PN alone in a study [23]. One of these studies was a prospective study of recipients of allo-HSCT after myeloablative conditioning [23], and the other was a retrospective study of recipients of myeloablative conditioning and reduced intensity conditioning [24]. Energy and protein intakes were not reported in these two studies and since the sickest patients are often those who cannot tolerate EN, their results may be considered as inconclusive.

In the present study, mean body weight decreased during the first year post-HSCT, in line with several other studies [3–6]. Moreover, mean percentage of total body water increased until 3 months and remained high compared to baseline during the 1-year follow-up. Thus, we speculate that weight gain (i.e. fluid retention) may be an indicator of a complication of allo-HSCT, and not a marker of improvement in nutritional status. An initial increase in fat-free mass and a loss of fat mass can be masking an increase in extracellular water in the early post-transplant period. This is partly in line with a small study showing fluid shifts to the extracellular space along with body cell mass loss till 30 days after allo-HSCT, where after body weight and fat mass remained constant [25]. Moreover, a RCT of old date reported an increase in fat mass and extracellular solids 28 days after allo-HSCT in patients receiving total parenteral nutrition compared to patients receiving an enteral feeding program [14]. In the present study fat-free mass and body water were stable after hospital discharge as opposed to loss of fat mass. This is partly in line with a study showing loss of both fat-free mass and fat mass 30 days after allo-HSCT [4]. Two of the present studies are of old date [14, 25] and differences in changes in body composition between studies may be due to the use of different assessment tools and the time points for measurements.

Weight change before allo-HSCT has been correlated with outcome [1, 2]. We found a positive association between BFMI and risk of death and NRM. In line with this has overweight before transplantation been associated with poor survival in allo-HSCT [20]. In contrast, weight loss before transplantation has been correlated to an increase in relapse rate after transplantation [1], but this was not confirmed in our study.

Weight gain may be a symptom or sign of one of the vascular endothelium syndromes (e.g. sinusoidal obstruction syndrome, capillary leak syndrome, engraftment syndrome) [9] activated by the conditioning regimen, cytokines produced by the injured tissues, endogenous microbial products translocated through damaged mucosal barriers [26], drugs [27, 28] and engraftment [9]. Moreover, fluid overload has been associated with poorer overall survival and increased NRM [8]. Additionally, glucocorticoid therapy is known to have an effect on weight gain and body composition, in particular muscle atrophy, central accumulation of fat mass and increased extracellular fluid volume [29, 30]. Corroborating this, one study found an association between decrease in indirect measurement of fat-free mass, and aGVHD, and glucocorticoid therapy [6]. We interpreted an early increase in FFMI and weight gain as fluid retention. Therefore our findings suggest that the sickest patients gained weight and this weight gain was a complication of HSCT. Former studies suggesting that malnutrition is an independent risk factor of mortality are cross-sectional and thus not able to establish causal relationships. It is questionable if weight loss is an independent risk factor that can be reversed with nutritional support or a result of the underlying disease [31, 32]. It is possible that weight loss prior to treatment not merely reflects insufficient nutritional intake and malnutrition, but also is a marker of disease severity.

Moreover, a significant association was found between weight gain and glucocorticoid usage from baseline to 6 weeks. However, independent of corticosteroid usage more patients (33.3%) experienced weight gain from baseline to 3 weeks than from baseline to 6 weeks (12.0%). An increase in total body water (i.e. fluid retention) in the early phase may be a symptom or sign of endothelium damage (organ dependent or systemic) due to toxicity of the conditioning regimen. An essential enzyme for metabolizing busulphan is glutathione transferase (GST), and increased toxicity of the conditioning regime may be a result of reduced activity of the GST enzymes [33]. An association between glutathione transferase *GSTA1* and *GSTM1* gene variants and busulphan pharmacokinetics and weight gain and mortality ≤30 days post-HSCT have been reported [7]. We can only speculate on genetics as an explanation model for the association between weight gain and death in the present study.

This study has several strengths and limitations. We achieved the recommended energy and protein intake in the intervention group [15]. Moreover, the energy and protein intake were based on the available recommendations, when we designed the study [22, 34] and are in line with current guidelines [13]. Furthermore, the targeted energy requirement was adjusted by resting energy expenditure measured with indirect calorimetry [35].

Dehydration or overhydration overestimate or underestimate fat-free mass or fat mass, respectively [36, 37]. A particular strength of our study was that we performed and interpreted longitudinally estimations of body composition with the same device under the same conditions [36, 37]. However, the main study was not powered for these secondary analyses.

In summary, we found no differences between the intervention and control group in risk of death, relapse, and NRM after 1 year. Moreover, changes in body weight and body composition were significant during 1-year follow-up, but similar in both groups. Weight gain was associated with increased risk of death and NRM during 1-year follow-up and increased risk of relapse after 3 months. In conclusion, weight gain is possibly due to fluid retention and is an indicator of complications in HSCT, rather than a marker of improved nutritional status.

## Supplementary information

Supplemental Information

## References

[1] Dietrich S, Radujkovic A, Stolzel F, Falk CS, Benner A, Schaich M (2015). Pretransplant metabolic distress predicts relapse of acute myeloid leukemia after allogeneic stem cell transplantation. Transplantation.

[2] Urbain P, Birlinger J, Ihorst G, Biesalski HK, Finke J, Bertz H (2013). Body mass index and bioelectrical impedance phase angle as potentially modifiable nutritional markers are independent risk factors for outcome in allogeneic hematopoietic cell transplantation. Ann Hematol.

[3] Fuji S, Mori T, Khattry N, Cheng J, Do YR, Yakushijin K (2015). Severe weight loss in 3 months after allogeneic hematopoietic SCT was associated with an increased risk of subsequent non-relapse mortality. Bone Marrow Transplant.

[4] Urbain P, Birlinger J, Lambert C, Finke J, Bertz H, Biesalski HK (2013). Longitudinal follow-up of nutritional status and its influencing factors in adults undergoing allogeneic hematopoietic cell transplantation. Bone Marrow Transplant.

[5] Rieger CT, Wischumerski I, Rust C, Fiegl M (2015). Weight loss and decrease of body mass index during allogeneic stem cell transplantation are common events with limited clinical impact. PLoS ONE.

[6] Brotelle T, Lemal R, Cabrespine A, Combal C, Hermet E, Ravinet A (2018). Prevalence of malnutrition in adult patients previously treated with allogeneic hematopoietic stem-cell transplantation. Clin Nutr.

[7] Bremer S, Floisand Y, Brinch L, Gedde-Dahl T, Bergan S (2015). Glutathione transferase gene variants influence busulfan pharmacokinetics and outcome after myeloablative conditioning. Ther Drug Monit.

[8] Rondon G, Saliba RM, Chen J, Ledesma C, Alousi AM, Oran B (2017). Impact of fluid overload as new toxicity category on hematopoietic stem cell transplantation outcomes. Biol Blood Marrow Transplant.

[9] Carreras E, Diaz-Ricart M (2011). The role of the endothelium in the short-term complications of hematopoietic SCT. Bone Marrow Transplant.

[10] Weisdorf SA, Lysne J, Wind D, Haake RJ, Sharp HL, Goldman A (1987). Positive effect of prophylactic total parenteral nutrition on long-term outcome of bone marrow transplantation. Transplantation.

[11] Mousavi M, Hayatshahi A, Sarayani A, Hadjibabaie M, Javadi M, Torkamandi H (2013). Impact of clinical pharmacist-based parenteral nutrition service for bone marrow transplantation patients: a randomized clinical trial. Support Care Cancer.

[12] Murray SM, Pindoria S. Nutrition support for bone marrow transplant patients. Cochrane Database Syst Rev. 2009;CD002920. 10.1002/14651858.CD002920.pub3 19160213

[13] Arends J, Bachmann P, Baracos V, Barthelemy N, Bertz H, Bozzetti F (2017). ESPEN guidelines on nutrition in cancer patients. Clin Nutr.

[14] Szeluga DJ, Stuart RK, Brookmeyer R, Utermohlen V, Santos GW (1987). Nutritional support of bone marrow transplant recipients: a prospective, randomized clinical trial comparing total parenteral nutrition to an enteral feeding program. Cancer Res.

[15] Skaarud KJ, Hjermstad MJ, Bye A, Veierod MB, Gudmundstuen AM, Lundin KEA (2018). Effects of individualized nutrition after allogeneic hematopoietic stem cell transplantation following myeloablative conditioning; a randomized controlled trial. Clin Nutr Espen.

[16] Bauer J, Capra S, Ferguson M (2002). Use of the scored patient-generated subjective global assessment (PG-SGA) as a nutrition assessment tool in patients with cancer. Eur J Clin Nutr.

[17] Cederholm T, Bosaeus I, Barazzoni R, Bauer J, Van Gossum A, Klek S (2015). Diagnostic criteria for malnutrition—an ESPEN Consensus Statement. Clin Nutr.

[18] Kyle UG, Schutz Y, Dupertuis YM, Pichard C (2003). Body composition interpretation. Contributions of the fat-free mass index and the body fat mass index. Nutrition.

[19] Andersen PK, Geskus RB, de Witte T, Putter H (2012). Competing risks in epidemiology: possibilities and pitfalls. Int J Epidemiol.

[20] Sorror ML, Maris MB, Storb R, Baron F, Sandmaier BM, Maloney DG (2005). Hematopoietic cell transplantation (HCT)-specific comorbidity index: a new tool for risk assessment before allogeneic HCT. Blood.

[21] Gratwohl A (2012). The EBMT risk score. Bone Marrow Transplant.

[22] Martin-Salces M, de Paz R, Canales MA, Mesejo A, Hernandez-Navarro F (2008). Nutritional recommendations in hematopoietic stem cell transplantation. Nutrition.

[23] Seguy D, Duhamel A, Rejeb MB, Gomez E, Buhl ND, Bruno B (2012). Better outcome of patients undergoing enteral tube feeding after myeloablative conditioning for allogeneic stem cell transplantation. Transplantation.

[24] Guieze R, Lemal R, Cabrespine A, Hermet E, Tournilhac O, Combal C (2014). Enteral versus parenteral nutritional support in allogeneic haematopoietic stem-cell transplantation. Clin Nutr.

[25] Cheney CL, Abson KG, Aker SN, Lenssen P, Cunningham BA, Buergel NS (1987). Body composition changes in marrow transplant recipients receiving total parenteral nutrition. Cancer.

[26] Eissner G, Multhoff G, Holler E (1998). Influence of bacterial endotoxin on the allogenicity of human endothelial cells. Bone Marrow Transplant.

[27] Fuste B, Mazzara R, Escolar G, Merino A, Ordinas A, Diaz-Ricart M (2004). Granulocyte colony-stimulating factor increases expression of adhesion receptors on endothelial cells through activation of p38 MAPK. Haematologica.

[28] Mercanoglu F, Turkmen A, Kocaman O, Pinarbasi B, Dursun M, Selcukbiricik F (2004). Endothelial dysfunction in renal transplant patients is closely related to serum cyclosporine levels. Transpl Proc.

[29] McKay LI, Cidlowski JA. Physiologic and pharmacologic effects of corticosteroids. In: Kufe D, Pollock R, Weichselbaum R, editors. Holland-Frei cancer medicine, 6th edn. Hamilton, ON: BC Decker; 2003.

[30] Hickson RC, Marone JR (1993). Exercise and inhibition of glucocorticoid-induced muscle atrophy. Exerc Sport Sci Rev.

[31] Baumgartner A, Bargetzi A, Zueger N, Bargetzi M, Medinger M, Bounoure L (2017). Revisiting nutritional support for allogeneic hematologic stem cell transplantation—a systematic review. Bone Marrow Transplant.

[32] Baumgartner A, Zueger N, Bargetzi A, Medinger M, Passweg JR, Stanga Z (2016). Association of nutritional parameters with clinical outcomes in patients with acute myeloid leukemia undergoing haematopoietic stem cell transplantation. Ann Nutr Metab.

[33] Czerwinski M, Gibbs JP, Slattery JT (1996). Busulfan conjugation by glutathione S-transferases alpha, mu, and pi. Drug Metab Dispos.

[34] Arends J, Bodoky G, Bozzetti F, Fearon K, Muscaritoli M, Selga G (2006). ESPEN guidelines on enteral nutrition: non-surgical oncology. Clin Nutr.

[35] Branson RD, Johannigman JA (2004). The measurement of energy expenditure. Nutr Clin Pract.

[36] Haverkort EB, Reijven PL, Binnekade JM, de van der Schueren MA, Earthman CP, Gouma DJ (2015). Bioelectrical impedance analysis to estimate body composition in surgical and oncological patients: a systematic review. Eur J Clin Nutr.

[37] Kyle UG, Bosaeus I, De Lorenzo AD, Deurenberg P, Elia M, Manuel Gomez J (2004). Bioelectrical impedance analysis-part II: utilization in clinical practice. Clin Nutr.

