# Supplementary material for: Body weight, body composition and survival after 1 year: follow-up of a nutritional intervention trial in allo-HSCT recipients
Source: Bone Marrow Transplant. 2019 Aug 27;54(12):2102–9. doi: 10.1038/s41409-019-0638-6 (PMC6957463; doi:10.1038/s41409-019-0638-6)
Supplement: Supplementary file 1 — Supplemental Information [file 41409_2019_638_MOESM1_ESM.docx]

Supplementary Information

**Body weight, body composition and survival after one year: follow-up of a nutritional intervention trial in allo-HSCT recipients**

| **Table S1.** Diagnoses and comorbidity scores at baseline by allocated treatment group^*^ | | | |
| --- | --- | --- | --- |
|  | Intervention | Control |  |
|  | (n= 57) | (n= 60) |  |
| Other diagnoses |  |  |  |
| ACML | 1 (2) | - |  |
| APL | 1 (2) | - |  |
| ABL | - | 1 (2) |  |
| AUL | - | 1 (2) |  |
| MCL | - | 1 (2) |  |
| MF | 2 (4) | - |  |
| MS | - | 1 (2) |  |
| HCTI – CI scores |  |  |  |
| 0 | 42 (73) | 45 (74) |  |
| 1 | 5 (9) | 9 (15) |  |
| 2 | 3 (5) | 1 (2) |  |
| 3 | 4 (7) | 4 (7) |  |
| 4 | 1 (2) | 1 (2) |  |
| 5 | 1 (2) | - |  |
| 6 | - | - |  |
| 7 | - | - |  |
| 8 | 1 (2) | - |  |
| EBMT scores |  |  |  |
| 0 | 2 (4) | 2 (3) |  |
| 1 | 3 (5) | 4 (7) |  |
| 2 | 11 (19) | 13 (22) |  |
| 3 | 17 (30) | 17 (28) |  |
| 4 | 14 (25) | 14 (23) |  |
| 5 | 6 (11) | 8 (14) |  |
| 6 | 3 (5) | 2 (3) |  |
| 7 | 1 (2) | - |  |
| ^*^Previously published [1]. Values are frequencies (%), *ACML* Atypical chronic myeloid leukemia, *APL* Acute promyelocytic leukemia, *ABL* Acute basophilic leukemia, *AUL* Acute undifferentiated leukemia, *MCL* Mast cell leukemia, *MF* Myelofibrosis, *MS* Myeloid sarcoma, *HCTI-CI* index Hematopoietic Cell Transplantation-specific comorbidity index, *EBMT* score European Group for Blood and Marrow Transplantation score. | | |  |

| **Table S2.** Body weight, fat-free mass and body fat mass index and total body water during one-year follow-up | | | | | | | |
| --- | --- | --- | --- | --- | --- | --- | --- |
|  |  | Intervention | | Control | | P-value^*^ | |
|  |  | n | Mean (SD) | n | Mean (SD) | Group effect | Time effect |
| Body weight (kg) | | | | | | | |
|  | Baseline | 57 | 77.7 (16.2) | 60 | 75.9 (15.2) |  |  |
|  | 3 weeks | 52 | 77.2 (15.3) | 59 | 75.0 (14.4) |  |  |
|  | 6 weeks | 50 | 73.4 (14.1) | 58 | 71.5 (13.8) |  |  |
|  | 3 months | 44 | 72.8 (14.6) | 52 | 70.6 (14.1) |  |  |
|  | 6 months | 39 | 69.8 (12.5) | 44 | 69.9 (13.9) |  |  |
|  | 9 months | 37 | 68.8 (12.8) | 40 | 68.7 (14.1) |  |  |
|  | 12 months | 37 | 68.9 (11.9) | 38 | 69.4 (14.5) | 0.17 | <0.001 |
| Fat-free mass index (kg/m^2^) | | | | | | | |
|  | Baseline | 56 | 18.5 (3.1) | 59 | 18.2 (2.5) |  |  |
|  | 3 weeks | 48 | 19.2 (3.1) | 55 | 19.1 (3.1) |  |  |
|  | 6 weeks | 38 | 18.1 (2.9) | 42 | 18.3 (2.9) |  |  |
|  | 3 months | 39 | 18.5 (3.0) | 48 | 18.5 (3.0) |  |  |
|  | 6 months | 38 | 18.2 (2.9) | 42 | 18.3 (2.7) |  |  |
|  | 9 months | 35 | 17.9 (2.8) | 36 | 18.3 (2.7) |  |  |
|  | 12 months | 34 | 18.1 (2.7) | 36 | 18.4 (2.8) | 0.56 | <0.001 |
| Body fat mass index (kg/m^2^) | | | | | | | |
|  | Baseline | 56 | 6.3 (3.1) | 59 | 6.2 (3.0) |  |  |
|  | 3 weeks | 48 | 5.6 (3.1) | 55 | 5.0 (3.0) |  |  |
|  | 6 weeks | 38 | 5.5 (2.9) | 42 | 4.6 (3.1) |  |  |
|  | 3 months | 39 | 4.7 (2.7) | 48 | 4.1 (2.7) |  |  |
|  | 6 months | 38 | 4.1 (2.5) | 42 | 4.4 (2.7) |  |  |
|  | 9 months | 35 | 4.3 (2.5) | 36 | 4.0 (2.2) |  |  |
|  | 12 months | 34 | 4.1 (2.5) | 36 | 4.3 (2.5) | 0.41 | <0.001 |
| Total body water (%) | | | | | | | |
|  | Baseline | 56 | 55.3 (6.7) | 59 | 55.2 (7.1) |  |  |
|  | 3 weeks | 48 | 57.4 (7.2) | 55 | 58.5 (7.9) |  |  |
|  | 6 weeks | 38 | 56.9 (7.1) | 42 | 59.0 (8.3) |  |  |
|  | 3 months | 39 | 60.3 (7.5) | 48 | 60.3 (7.5) |  |  |
|  | 6 months | 38 | 60.2 (7.1) | 42 | 59.7 (6.9) |  |  |
|  | 9 months | 35 | 59.6 (7.3) | 36 | 60.9 (6.1) |  |  |
|  | 12 months | 34 | 60.2 (7.4) | 36 | 60.0 (6.8) | 0.98 | <0.001 |
| ^*^P-values from mixed model analysis. | | | | | | | |

| **Table S3.** Hazard ratios (HRs) and 95% confidence intervals (CIs) for treatment group, body weight, fat-free mass index and body fat mass index, and risk of death post-discharge. | | | | | | | |
| --- | --- | --- | --- | --- | --- | --- | --- |
|  |  | Univariable | | |  | Multivariable^*^ | |
| Variables | N | Cases | HR (95% CI) | P-value |  | HR (95% CI) | P-value |
| Discharge as time-varying variable added in the model |  |  |  |  |  |  |  |
| Weight (kg) | 117 | 35 | 1.03 (1.00-1.05) | 0.02 |  | 1.04 (1.01-1.06) | 0.01 |
| FFMI (kg/m^2^) | 115 | 35 | 1.04 (0.93-1.17) | 0.45 |  | 1.06 (0.90-1.25) | 0.50 |
| BFMI (kg/m2) | 115 | 35 | 1.16 (1.04-1.30) | 0.01 |  | 1.22 (1.08-1.38) | 0.01 |
| *FFMI* Fat-free mass index, *BFMI* body fat mass index, *NRM* non-relapse mortality, ^*^Adjusted for treatment group, gender and age. | | | | | | | |

| **Table S4a.** Hazard ratios (HRs) and 95% confidence intervals (CIs) for body weight, fat-free mass index and body fat mass index at baseline^*^ and risk of death, relapse and non-relapse mortality | | | | | | | |
| --- | --- | --- | --- | --- | --- | --- | --- |
|  |  | Univariable | | |  | Multivariable^**^ | |
|  | N | Cases | HR (95% CI) | P-value |  | HR (95% CI) | P-value |
| Death |  |  |  |  |  |  |  |
| Weight (kg) | 117 | 35 | 1.01 (0.99-1.03) | 0.23 |  | 1.02 (0.99-1.04) | 0.21 |
| FFMI (kg/m^2^) | 115 | 35 | 1.02 (0.90-1.14) | 0.78 |  | 1.00 (0.84-1.20) | 0.97 |
| BFMI (kg/m2) | 115 | 35 | 1.11 (0.99-1.24) | 0.07 |  | 1.13 (1.00-1.27) | 0.04 |
| Relapse |  |  |  |  |  |  |  |
| Weight (kg) | 117 | 20 | 1.01 (0.98-1.03) | 0.62 |  | 1.00 (0.96-1.03) | 0.77 |
| FFMI (kg/m^2^) | 115 | 20 | 1.07 (0.91-1.24) | 0.42 |  | 0.96 (0.78-1.19) | 0.74 |
| BFMI (kg/m^2^) | 115 | 20 | 0.94 (0.80-1.11) | 0.48 |  | 0.98 (0.83-1.16) | 0.83 |
| NRM |  |  |  |  |  |  |  |
| Weight (kg) | 117 | 20 | 1.02 (0.99-1.04) | 0.21 |  | 1.03 (0.99-1.06) | 0.14 |
| FFMI (kg/m^2^) | 115 | 20 | 1.01 (0.87-1.18) | 0.88 |  | 1.02 (0.79-1.31) | 0.90 |
| BFMI (kg/m^2^) | 115 | 20 | 1.16 (1.01-1.35) | 0.04 |  | 1.19 (1.02-1.39) | 0.03 |
| *FFMI* Fat-free mass index, *BFMI* body fat mass index, *NRM* non-relapse mortality, ^*^Modeled as continuous variables, ^**^Adjusted for treatment group, gender and age. | | | | | | | |

| **Table S4b.** Hazard ratios (HRs) and 95% confidence intervals (CIs) for weight change before baseline and risk of death, relapse and non-relapse mortality | | | | | | | |
| --- | --- | --- | --- | --- | --- | --- | --- |
|  |  | Univariable | | |  | Multivariable^*^ | |
|  | N | Cases | HR (95% CI) | P-value |  | HR (95% CI) | P-value |
| Death |  |  |  |  |  |  |  |
| No weight loss^**^ | 47 | 13 | Ref. |  |  | Ref. |  |
| >0%-<5% loss | 27 | 10 | 1.43 (0.63-3.25) | 0.40 |  | 1.40 (0.61-3.24) | 0.43 |
| 5%-<10% loss | 21 | 4 | 0.68 (0.22-2.08) | 0.50 |  | 0.66 (0.21-2.06) | 0.48 |
| >10% loss | 22 | 8 | 1.40 (0.58-3.38) | 0.45 |  | 1.43 (0.58-3.50) | 0.43 |
| Relapse |  |  |  |  |  |  |  |
| No weight loss^**^ | 47 | 8 | Ref. |  |  | Ref. |  |
| >0%-<5% loss | 27 | 3 | 0.68 (0.18-2.55) | 0.56 |  | 0.66 (0.17-2.50) | 0.54 |
| 5%-<10% loss | 21 | 4 | 1.12 (0.34-3.71) | 0.86 |  | 1.26 (0.37-4.21) | 0.71 |
| >10% loss | 22 | 5 | 1.47 (0.48-4.50) | 0.50 |  | 1.25 (0.40-3.88) | 0.70 |
| NRM |  |  |  |  |  |  |  |
| No weight loss^**^ | 47 | 8 | Ref. |  |  | Ref. |  |
| >0%-<5% loss | 27 | 7 | 1.57 (0.57-4.34) | 0.38 |  | 1.61 (0.57-4.54) | 0.37 |
| 5%-<10% loss | 21 | 2 | 0.56 (0.12-2.64) | 0.46 |  | 0.53 (0.11-2.58) | 0.44 |
| >10% loss | 22 | 3 | 0.84 (0.22-3.16) | 0.79 |  | 0.94 (0.25-3.64) | 0.93 |
| *NRM* non-relapse mortality, ^*^Adjusted for treatment group, gender and age, ^**^The first category includes the patients that gained weight. | | | | | | | |

| **Table S5.** Number of patients (%) with no weight gain/weight loss versus weight gain and glucocorticoid usage in the early phase | | | | | | | | | | | |
| --- | --- | --- | --- | --- | --- | --- | --- | --- | --- | --- | --- |
|  | Baseline to three weeks | | |  | Three to six weeks | | |  | Baseline to six weeks | | |
|  | Glucocorticoid | | |  | Glucocorticoid | | |  | Glucocorticoid | | |
|  | Yes | No | P-value |  | Yes | No | P-value |  | Yes | No | P-value |
| No weight gain/weight loss | 11 (68.7) | 63 (66.3) |  |  | 28 (80.0) | 64 (87.7) |  |  | 26 (74.3) | 69 (94.5) |  |
| Weight gain | 5 (31.3) | 32 (33.7) | 0.85 |  | 7 (20.0) | 9 (12.3) | 0.29 |  | 9 (25.7) | 4 (5.5) | <0.004 |

**Reference**

1. Skaarud KJ, Hjermstad MJ, Bye A, Veierod MB, Gudmundstuen AM, Lundin KEA*,* et al. Effects of individualized nutrition after allogeneic hematopoietic stem cell transplantation following myeloablative conditioning; a randomized controlled trial. Clinical nutrition ESPEN. 2018;28:59-66.
